# Timeliness of Childhood Vaccinations in Kampala Uganda: A Community-Based Cross-Sectional Study

**DOI:** 10.1371/journal.pone.0035432

**Published:** 2012-04-23

**Authors:** Juliet N. Babirye, Ingunn M. S. Engebretsen, Frederick Makumbi, Lars T. Fadnes, Henry Wamani, Thorkild Tylleskar, Fred Nuwaha

**Affiliations:** 1 School of Public Health, Makerere University College of Health Sciences, Kampala Uganda; 2 Centre for International Health, University of Bergen, Bergen, Norway; 3 Department of Child and Adolescent Psychiatry, Haukeland University Hospital, Bergen, Norway; Laboratory of Malaria Immunology and Vaccinology, United States of America

## Abstract

**Background:**

Child survival is dependent on several factors including high vaccination coverage. Timely receipt of vaccines ensures optimal immune response to the vaccines. Yet timeliness is not usually emphasized in estimating population immunity. In addition to examining timeliness of the recommended Expanded Programme for Immunisation (EPI) vaccines, this paper identifies predictors of untimely vaccination among children aged 10 to 23 months in Kampala.

**Methods:**

In addition to the household survey interview questions, additional data sources for variables included data collection of child's weight and length. Vaccination dates were obtained from child health cards. Timeliness of vaccinations were assessed with Kaplan–Meier time-to-event analysis for each vaccine based on the following time ranges (lowest–highest target age): BCG (birth–8 weeks), polio 0 (birth–4 weeks), three polio and three pentavalent vaccines (4 weeks–2 months; 8 weeks–4 months; 12 weeks–6 months) and measles vaccine (38 weeks–12 months). Cox regression analysis was used to identify factors associated with vaccination timeliness.

**Results:**

About half of 821 children received all vaccines within the recommended time ranges (45.6%; 95% CI 39.8–51.2). Timely receipt of vaccinations was lowest for measles (67.5%; 95% CI 60.5–73.8) and highest for BCG vaccine (92.7%: 95% CI 88.1–95.6). For measles, 10.7% (95% CI 6.8–16.4) of the vaccinations were administered earlier than the recommended time. Vaccinations that were not received within the recommended age ranges were associated with increasing number of children per woman (adjusted hazard ratio (AHR); 1.84, 95% CI 1.29–2.64), non-delivery at health facilities (AHR 1.58, 95% CI 1.02–2.46), being unmarried (AHR 1.49, 95% CI 1.15–1.94) or being in the lowest wealth quintile (AHR 1.38, 95% CI 1.11–1.72).

**Conclusions:**

Strategies to improve vaccination practices among the poorest, single, multiparous women and among mothers who do not deliver at health facilities are necessary to improve timeliness of vaccinations.

## Introduction

Vaccine preventable diseases account for about a quarter of the 8 million deaths occurring annually among children under five years of age especially in low-income countries such as Uganda [1], [2]. Vaccination of children could therefore prevent more than 2 million child deaths each year thus increasing child survival [3] with its attendant economic gains [4], [5].

As more ambitious goals for immunisation and disease control are set in response to the declaration of the decade of vaccines such as introduction of established and new vaccines mainly in developing countries, pressures to meet short-term goals need to be balanced with substantial efforts to establish and sustain strong health systems for vaccine delivery, surveillance, and monitoring [2]. This is particularly important in Uganda where a child receives nine vaccine doses for complete vaccination and there are plans to introduce other vaccines such as the pneumococcal vaccine.

Previous interventions in Uganda focused on promoting high vaccination coverage. However, some studies show that high vaccination coverage rates for individual vaccines do not necessarily imply timely vaccination or population immunity [6], [7], [8]. Yet some sub-Saharan African countries report less than optimal vaccination coverage rates and some including the district health office in Kampala report coverage rates above 100% [9], and still record epidemics for diseases such as measles [10]. Assessment of the many challenges now and in the long term [11] is imperative for the success of the immunisation programme. This study examined timeliness for each vaccine in the expanded programme on immunisation (EPI) in Kampala and the factors that influence untimely vaccinations.

## Methods

### Study setting

The study was conducted in Kampala from June to September 2010. Kampala is the capital and largest urban area in Uganda. It covers approximately 200 km^2^ with a population density of more than 7400 persons/k m^2^ and a total population of about 1.6 million people. Salaried employees constitute 52% of the population. The annual population growth rate in Kampala is 3.8% and about 60% of this growth is by immigration into the city. Children below 5 years alone constitute 20% of the total population.

Although Kampala records the lowest childhood mortality rates in Uganda, the district still experienced a high infant mortality rate of 54 deaths per 1000 live births in 2006 [12]. Health services in Kampala are provided by government, non-governmental organization (NGO), and privately owned health facilities. All the government and NGO health facilities provide routine immunisation services in addition to outreach services.

The city is administratively divided into 5 divisions and each division is semi-autonomous with a separate work plan and budget. Three of the divisions; Central, Kawempe, and Rubaga, are better served with public health facilities. Nakawa and Makindye divisions are relatively least served by public health facilities and immunisation services. This study was conducted in Nakawa and Makindye divisions which contribute almost half (46%) of the total population in Kampala.

### Sample size calculation

The required sample size was 812 households using the formula by Bennnet et al [13] for cluster surveys with the following assumptions; a two-sided test with a precision of 0.03, 80% power, 7 households per cluster, intraclass correlation of 0.1, design effect of 1.6, proportion of those with complete vaccinations of 47% and a non-response rate of 37% (estimated among children aged 12–23 months with missing child health cards) [12].

### Eligibility and Sampling

Caretaker-child pairs were eligible for study inclusion if they were from households with a child aged 10 months to 23 months and if they had a child health card. Those without cards were excluded from full data collection to reduce recall bias in relation to dates of vaccination. The study team captured basic demographic data on those without child health cards to describe to which degree they shared characteristics with the study population for timeliness. One child per household was selected for study inclusion. If there were more than one eligible child per household such as twins in the house a coin was flipped to select one of them for study inclusion. Study participants were selected from 10 of 44 parishes in the two divisions.

A two stage sampling technique was employed for the selection of study participants. In the first stage 5 parishes were randomly selected from Nakawa division and 5 from Makindye division by use of computer generated random numbers. The number of respondents at each parish was determined using sampling proportionate to infant population size estimated for each parish using the population projection for 2010 from the Uganda Bureau of Statistics [14]. All local council-ones (LC-1; lowest administrative units) in the selected parishes were included in the study and the number of households for study inclusion per LC-1 was estimated as an average of the total number required in each parish.

At the second stage selection of households to be interviewed was conducted in the following manner; a random starting point in each LC-1 was identified, preferably a junction in the LC-1. Then beginning with the house on the eastern side, the data collectors moved house to house in clockwise concentric circles looking for eligible participants till the appropriate sample for that LC-1 was obtained.

In case a respondent in a selected household did not have an eligible child, declined to participate, was less than 18 years of age or was not home the next household was considered for study inclusion.

### Data Collection, Measurements and Handling

Time to vaccination for each EPI vaccine was obtained from vaccination dates and dates of birth which were noted down from child health cards. Most vaccinations were dated on the child health cards, but some vaccinations were recorded as “given” on the child health cards but the dates at which these vaccines were given was not registered. For these particular vaccines the age at vaccination for the same vaccine of the preceding child in the database was assumed for the missing age at vaccination. This was done to maintain the random distribution of ages at vaccination that is close to that in the population. This method was meant to give a more accurate result than would the assumption that the vaccination experience of the children with missing dates was the same. In addition, data on social, demographic, economic characteristics, nutritional status of the child and vaccination practices were collected using an interviewer administered structured questionnaire.

Weight and recumbent length were measured according to WHO standardized techniques [15]. Undressed or lightly clothed infants were weighed to the nearest 0.1 kg using 25 kilogram (kg) portable Salter spring scales and recumbent length was measured to the nearest 0.1 cm using a length board. Validation of weighing scales was done on a daily basis using standard weights.

Children's nutritional status was assessed by evaluating anthropometric data using the z-scores for weight-for-length (WLZ), length-for-age (LAZ) and weight-for-age (WAZ) using the WHO Child Growth Standards [16]. Wasting was defined as WLZ less than -2, stunting as LAZ less than -2 and underweight as WAZ less than -2 [15].

The household wealth index was developed by use of principal components analysis [17] with variables on asset ownership (radio, telephone, television, refrigerator, cupboard, bicycle, motorcycle, car/truck); materials of the dwelling structure (floor, wall, roof); availability of electricity, water and sanitation services; how many rooms in the house; and house ownership. The first component explained 30.9% of the variance. Regression factor scores generated from the first principal component were ranked in ascending order and then categorised into quintiles (1) poorest, to (5) least poor

Mobile phones were used to collect the data. The questionnaire was designed and managed with OpenXdata version 1.3.4 (http://www.openxdata.org). The questionnaire was downloaded onto mobile telephones. The collected data on the mobile telephones were then directly synchronized onto a database on a daily basis via internet. The data saved on the server was exported to an excel work sheet and then to SPSS version 17 (SPSS Inc. Chicago, Illinois) for analysis.

### Data analysis

According to the Ugandan National Expanded Programme on Immunisation (UNEPI), a child is considered fully vaccinated if it has received one dose of BCG (given at birth), four doses of polio vaccines (given at birth, 6 weeks, 10 weeks and 14 weeks), three doses of DPT, Hepatitis B, *H. influenzae* type b vaccine (given at 6 weeks, 10 weeks and 14 weeks), and one dose of the measles vaccine (given at 9 months). Timeliness of vaccinations was defined for each vaccine using the following WHO recommended time ranges: BCG (birth–8 weeks), polio 0 (birth–4 weeks), three polio and three pentavalent vaccines (4 weeks–2 months; 8 weeks–4 months; 12 weeks–6 months) and measles vaccine (38 weeks–12 months) [18].

Timeliness was analysed with Kaplan–Meier time-to-event analysis. An event was defined as not receiving a scheduled vaccine within the recommended time range . A child was censored if the scheduled vaccine was received within the recommended time range. Person-months of observation were estimated as time spent during the vaccine eligibility period up to when the vaccine was received either; within the recommended time range or earlier or past the recommended time but within the 23 months (thus censoring). If a child missed a preceding vaccination dose, then this child was not eligible to the follow-up of the subsequent vaccination dose. Only children with previous vaccination would be followed up for the subsequent vaccination, and remained in this analysis. For example, for a child to enter the risk set for measles he/she should have received the first set of vaccines before 38weeks. Thus, there were different entry points according to the different vaccines but entrance into the risk set occurred only once. Both the event and censoring were composite variables. A composite ordinal variable with all EPI vaccines in one variable was created with the first break in the schedule being taken as the time to event for each child. The event status was assigned to each individual vaccine using the recommended WHO ranges and censoring in the composite variable therefore assumed the status of the vaccine at which the first break in the vaccination schedule was observed.

Cox regression analysis was used to examine the factors associated with failure to vaccinate on time using the ordinal variable. Factors that were statistically significant at univariate analysis were entered into a multivariate model. Since, child vaccination is highly dependent on the health seeking behaviour of the child caretakers, therefore the characteristics of the child caretakers were considered in constructing the multivariate model. Child characteristics such as child morbidity and nutritional status were also considered for model construction because the most critical period for growth, health and development is from birth to two years. This is also the period most marked by childhood illnesses and acute respiratory infections [12]. Malnutrition increases the risk of these illnesses. This ill health could in turn prevent the mothers from taking children for vaccinations on the allocated dates, thus leading to untimely vaccinations. Maternal age was not included in the multivariate model since it was highly correlated with the number of siblings each child had (Pearson correlation = 0.56, p<0.001). The number of siblings each child had was considered a better proxy indicator for maternal experience in child care than maternal age. No other variables were strongly correlated with each other.

Model robustness was checked by Wald chi square. Cluster sampling was adjusted for in all analyses using complex samples analysis employing the probability proportional to size sampling method.

### Ethics

Ethics approval was obtained from Makerere University School of Public Health Higher Degrees Research and Ethics Committee (IRB00005876FWA/Protocol 085) and independently from the Uganda National Council for Science and Technology (HS 786). Study participants provided informed written consent to the interview and to collection of the child's anthropometric measurements.

## Results

Nine hundred eligible households were approached for study inclusion. Seventy nine (79/900, 8.8%) were excluded from full data collection due to misplaced or lost cards (72.2%, 57/79), child health cards had been destroyed by fire or eaten by rats (7.6%, 6/79), children had never been immunised (12.7%, 10/79), declined study participation (7.6%, 6/79). We included 821 study subjects in full data collection and in data analysis. Almost all respondents were mothers of the eligible child (95.6%, 785/821). Other study respondents included fathers, mother's mother, or other female relatives.

The mean age for respondents included in full data collection was 25.6 years (95% CI 24.3–27.0) and 25.8 years (95% CI 25.5–26.2) for those excluded. The mean children's age for those included in the study was 16.3 months (95% CI 16.0–16.6) and 15.9 months (95% CI 15.1–16.7) for those excluded. Less than half of those included in the study (41.8%, 95% CI 38.3–45.2) and a third of those excluded (30.3%, 95% CI 19.9–40.7) had secondary school education. Lower proportions of those included (21.6%, 95% CI 18.8–24.5) and excluded from the study (13.2%, 95% CI 5.5–20.8) had tertiary education.

Overall 77.2% (95% CI 74.3%–80.0%) of 821 children were fully vaccinated. Receipt of vaccinations ranged from 80.6% (95% CI 77.6–83.3) for measles to 99.0% (95% CI 98.4–99.7) for BCG vaccine (Table 1). Among those that had not received the measles vaccine 3.8% (31/821) had not reached their first birthday. Vaccinations that were recorded as given but not dated ranged from 1.6% (95% CI 0.7–2.4) for Polio 2 to 4.8% (95% CI 3.4–6.3) for polio 0.

**Table 1:** Number vaccinated, missing dates and timeliness of each vaccine among 821 children with child health cards.

Vaccine	Number vaccinated	Missing vaccination dates	Timeliness of each vaccine		
	n = 821 (%)	% (95% CI)	Timely (%, 95% CI)	Late (%, 95% CI)	Early (%, 95% CI)
BCG	813 (99.0)	4.4 (3.0–5.8)	92.7 (88.1–95.6)	7.3 (4.4–11.9)	0.0 (0.0-0.0)
Polio 0	795 (96.8)	4.8 (3.4–6.3)	84.8 (78.9–89.3)	15.2 (10.7–21.1)	0.0 (0.0-0.0)
Polio 1	798 (97.2)	2.5 (1.5–3.6)	71.4 (65.0–77.1)	25.9 (19.7–33.3)	2.7 (1.8–3.9)
Polio 2	772 (94.1)	1.6 (0.7–2.4)	78.3 (74.1–82.0)	21.1 (17.7–25.7)	0.6 (0.2–1.6)
Polio 3	730 (89.0)	2.5 (1.5–3.6)	74.9 (70.4–78.9)	24.6 (20.4–29.4)	0.5 (0.2–1.4)
Pentavalent 1	805 (98.1)	2.3 (1.3–3.3)	72.4 (66.0–78.0)	25.7 (19.7–32.7)	1.9 (1.4–2.8)
Pentavalent 2	770 (93.8)	1.7 (0.8–2.6)	77.8 (72.8–82.1)	21.8 (17.4–27.1)	0.4 (0.1–1.1)
Pentavalent 3	731 (89.1)	2.3 (1.3–3.3)	74.9 (69.8–79.3)	24.9 (20.4–30.0)	0.2 (0.1–0.8)
Measles	663 (80.6)	4.4 (3.0–5.8)	67.5 (60.5–73.8)	21.8 (18.3–25.8)	10.7 (6.8–16.4)

### Timeliness

Timely vaccinations ranged from 67.5% (95% CI 60.5–73.8) for measles vaccine to 92.7% (95% CI 88.1–95.6)S for BCG vaccine (table 1). For measles 10.7% (95% CI 6.8–16.4) of the vaccinations were given early. There was a statistically significant difference between timely receipt of BCG (92.7%, 95% CI 88.1%–95.6%) and polio 0 (84.8%, 95% CI 78.9–89.3%).

**Figure 1:**
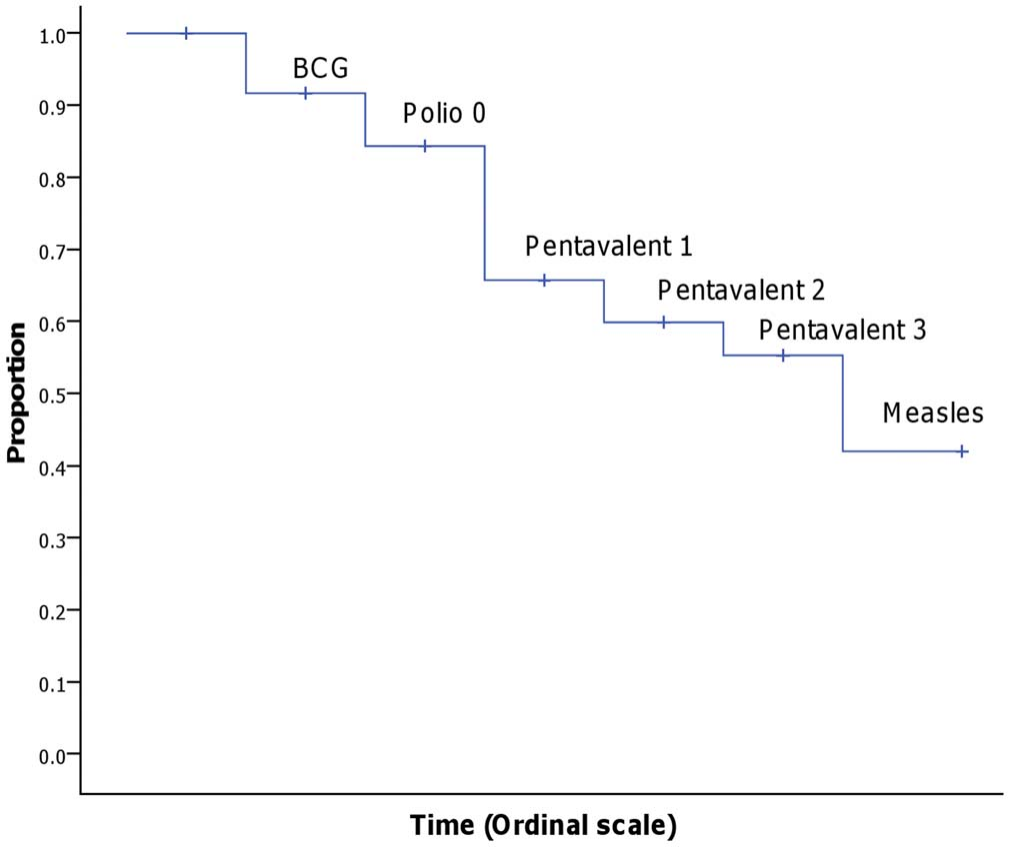
Proportion of timely vaccinations by child's age. The figure illustrates the proportion that received the vaccines within the recommended time ranges for all vaccines. The line drops represent the proportion that received all the vaccines to that point within the recommended time ranges, but does not get the given vaccine within the recommended range (e.g. if a child received all vaccines within the recommend time except the measles vaccine, the child will add to the line drop at measles). The steps are ordinal and do not depict the actual length of time within each time step.

Overall, less than half (45.6%, 95% CI 39.8–51.2) of all children (374/821) received all vaccines within the recommended time ranges (figure 1). We conducted a sensitivity analysis in which we either included or excluded the 79 cases that were excluded from full data collection due to lack of child health cards. If we assumed that these 79 had untimely vaccinations then the proportion of those that were fully timely decreases to 41.6% (374/900). And if we assumed that the 79 were timely for all vaccines then the proportion of those that were fully timely increases to 50.3% (374+79 = 453/900).

### Predictors of untimely vaccination

Predictors of untimely vaccinations at univariate and multivariate level are shown in table S1. In the multivariate model, untimely vaccination increased with increasing number of children. It was higher among those that did not deliver at a hospital, were unmarried, and among those in the lowest wealth quintile.

Untimely vaccinations decreased with increasing maternal or paternal education at univariate analysis but this relationship disappeared in the adjusted model. Maternal age, paternal age and nutritional status of the child were unrelated to timely vaccination. The adjusted model predicted timing of vaccination well (Model χ^2^ = 76; df = 9; p<0.001).

## Discussion

About half of all children received all vaccinations in a timely manner and 11% of measles vaccinations were received earlier than the recommended age. Untimely vaccination was more likely if there were more than one child in the household, the child was born outside a hospital, the child's household was among the poorest, and the respondent was unmarried.

Higher rates of untimely vaccinations have been reported in other study settings [7], [19], [20]. The implication of delay in receipt of vaccines is that a pool of children with incomplete or no immunisation may build up. The presence of such a pool of susceptible children predisposes to outbreaks of vaccine preventable diseases [21]. These outbreaks occur when the epidemic threshold is exceeded and this may occur much faster when poor vaccine timeliness is coupled with low rates of vaccination coverage and low vaccine effectiveness [22].

A tenth of children in this study received measles vaccination earlier than the recommended age. Similar proportions are reported by other researchers [7]. Early vaccinations have administrative, programmatic, and cost implications. The early vaccinations contribute to overall coverage figures leading to an overestimation of actual population immunity. Measles doses that are given early to be considered valid must be repeated, which results in unnecessary risk for adverse reaction and more complex immunisation schedules for child care takers [20]. None of the children in our study received booster measles doses. The current Ugandan immunisation schedule provides for only one measles vaccine at 9 months of the infant's age. However, discussions are ongoing for a two dose schedule. With the addition of three new vaccines namely; pneumococcal vaccine (scheduled roll out early 2012), rotavirus vaccine (end of 2012) and human papilloma virus vaccine (not yet scheduled), the EPI schedule is set to be even more complicated. Therefore assessing timeliness of the vaccinations regularly is even more critical for the success of the EPI programme.

This study identified societal factors associated with timely vaccination. Other studies have shown that maternal education, attendance for antenatal care, and parity are associated with better utilisation of child vaccination services [23], [24], [25]. In this study, women who received antenatal care were not significantly more likely to have better timely vaccination for their child compared to those who did not. This may be attributed to the fact that antenatal care attendance is almost universal in this setting [12].

On the other hand, delivery at the health facility predicts better timely vaccinations as has been reported from other study settings [23], [25]. It is possible that mothers who deliver at health facilities may be more frequent users of health facilities and services including immunisation for children. The administration of BCG and polio at birth is required for registered maternity health facilities and may partly account for better timely vaccination of BCG and Polio 0 than the subsequent vaccines in the EPI schedule.

Respondents in the poorest quintile were most likely to have untimely vaccinations. This complements our qualitative findings which indicated that poverty related factors hindered utilisation of immunisation services [26]. The fact that maternal education was not an independent predictor for timely vaccination in this analysis indicates that poverty in this setting is a more important determinant of timely vaccinations than maternal education. In our study more than 60% of all mothers had secondary school or higher education unlike reports from rural settings with around 30% in this category [7], [24].

Children with several siblings were more likely to have untimely vaccinations. This relationship has been reported by other researchers and has been linked to the higher cost and demands on resources caused by having more children in a household and this may adversely affect healthcare utilization [23], [27], [28]. Furthermore, vaccination of children protects against an unseen threat and the benefits of these activities are not immediately apparent, thus there is very little motivation for child caretakers to prioritize vaccination services amidst competing demand for time [29].

### Methodological considerations

This study was conducted in Kampala which consists of urban and peri-urban areas. Our results therefore may have implications for vaccination programmes in similar settings of Sub-Saharan Africa. Respondents without a child health card were not included in this study and could have led to biased sampling. They generally had less education than the respondents included in full data collection. It is likely that those without cards had partially immunised their children, had never immunised or had more untimely vaccinations compared to those with cards. Consequently, the level of timely vaccination in Kampala may be lower than what is reported in this study. Therefore one of the issues for further research on child population immunity is to identify the best source of accurate information on vaccination status without resorting to invasive procedures.

A potential bias was introduced in the study especially in estimation of timeliness for measles vaccine since children from 10 months old were included in this study. In Uganda all children should get the measles vaccine at 9 months but WHO recommends receipt between 38 weeks to 12 months. Therefore 4% of the children in this study that had not yet reached their first birthday and had not received measles vaccine were late according to the Ugandan schedule but were not late according to the WHO recommended end point for measles. In this study such children were censored during analysis based on WHO guidelines with consequent overestimation of timely vaccination for measles.

### Conclusions

Most studies that report on child utilization services usually focus on the number of vaccinations accumulated by specified ages. Our analysis of timeliness of vaccination in this setting shows that children rarely receive all vaccinations as recommended. Ministries of health should use timeliness of vaccinations along with other measures to determine children's susceptibility to vaccine-preventable diseases and to evaluate the quality of vaccination programs.

In a previous qualitative report we concluded that mothers needed additional support in order to utilise immunisation services [26]. This quantitative study has identified the specific categories of mothers that require this additional support. Strategies to improve utilisation of vaccination services for these high risk groups are urgently needed.

## Supporting Information

Table S1 Predictors of untimely vaccinations at univariate and multivariate level are shown. The multivariate model included child characteristics, caretaker characteristics, and distal determinants for health such as wealth status.
(DOC)
