# Supplementary material for: Timeliness of Childhood Vaccinations in Kampala Uganda: A Community-Based Cross-Sectional Study
Source: PLoS One. 2012 Apr 23;7(4):e35432. doi: 10.1371/journal.pone.0035432 (PMC3335141; doi:10.1371/journal.pone.0035432)
Supplement: Table S1 — Predictors of untimely vaccinations at univariate and multivariate level are shown. The multivariate model included child characteristics, caretaker characteristics, and distal determinants for health such as wealth status. (DOC) [file pone.0035432.s001.doc]

Table S1: Predictors of untimely vaccination using Cox-regression analysis (cluster adjusted)

| **Variable** | **n=821 (%)** | **Unadjusted HR (95% CI)** | **Adjusted HR (95% CI)** |
| --- | --- | --- | --- |
| **Age of child (months)** |  |  |  |
| 10-11 | 102 (12.4) | 0.78 (0.51-1.21) |  |
| 12-17 | 392 (47.7) | 0.93 (0.70-1.24) |  |
| 18-23 | 327 (39.9) | 1 |  |
| **Gender of the child** |  |  |  |
| Boy | 432 (52.6) | 0.92 (0.77-1.09) |  |
| Girl | 389 (47.4) | 1 |  |
| **Number of siblings** |  |  |  |
| None | 283 (34.6) | 1 | 1 |
| One | 249 (30.4) | 1.36 (0.98-1.88) | 1.45 (1.07-1.98) |
| 2-3 | 220 (26.9) | 1.34 (0.94-1.91) | 1.55 (1.11-2.18) |
| ≥4 | 67 (8.1) | 1.75 (1.12-2.74) | 1.84 (1.29-2.64) |
| **Did you attend antenatal care during the pregnancy of this child?** | | |  |
| Yes | 807 (98.3) | 1 |  |
| No | 14 (1.7) | 1.50 (0.58-3.90) |  |
| **Place of delivery** |  |  |  |
| Hospital | 520 (63.4) | 1 | 1 |
| Health centre | 243 (29.6) | 1.27 (1.06-1.53) | 1.29 (0.99-1.68) |
| Home | 57 (7.0) | 1.85 (1.31-2.62) | 1.58 (1.02-2.46) |
| **Reported fever in two weeks prior to interview** |  |  |  |
| No | 591 (72.0) | 1 |  |
| Yes | 230 (28.0) | 1.13 (0.87-1.45) |  |
| **Reported cough in two weeks prior to interview** | |  |  |
| No | 481 (58.7) | 1 |  |
| Yes | 339 (41.3) | 1.06 (0.78-1.42) |  |
| **Reported diarrhoea in two weeks prior to interview** | |  |  |
| No | 671 (81.8) | 1 |  |
| Yes | 149 (18.2) | 0.93 (0.71-1.23) |  |
| **Child wasted** |  |  |  |
| No | 791 (98.9) | 1 |  |
| Yes | 9 (1.1) | 1.73 (0.88-3.41) |  |
| **Child stunted** |  |  |  |
| No | 582 (72.3) | 1 | 1 |
| Yes | 223 (27.7) | 1.36 (1.16-1.60) | 1.17 (0.79-1.75) |
| **Child underweight** |  |  |  |
| No | 756 (94.5) | 1 | 1 |
| Yes | 44 (5.5) | 1.54 (1.03-2.29) | 1.15 (0.74-1.78) |
| **Mother’s age (years)** |  |  |  |
| 18-24 | 409 (47.4) | 1 |  |
| 25-29 | 266 (31.5) | 0.84 (0.69-1.01) |  |
| 30-34 | 115 (13.7) | 1.16 (0.84-1.61) |  |
| ≥35 | 66 (7.4) | 1.15 (0.84-1.56) |  |
| **Mother’s education** |  |  |  |
| Stopped in primary/none | 154 (19.2) | 2.13 (1.56-2.89) | 1.17 (0.78-1.75) |
| Completed primary | 139 (17.4) | 1.68 (1.34-2.11) | 1.06 (0.71-1.58) |
| Secondary | 334 (41.8) | 1.34 (1.03-1.75) | 1.00 (0.69-1.44) |
| Higher education | 173 (21.6) | 1 | 1 |
| **Father’s education** |  |  |  |
| Primary | 83 (12.0) | 2.08 (1.31-3.30) | 1.27 (0.93-1.73) |
| Secondary | 271 (39.0) | 1.41 (1.06-1.89) | 1.10 (0.87-1.38) |
| Higher education | 340 (49.0) | 1 | 1 |
| **Father’s age (years)** |  |  |  |
| ≤29 | 283 (39.5) | 0.92 (0.70-1.20) |  |
| 30-34 | 199 (27.8) | 0.78 (0.53-1.14) |  |
| ≥35 | 234 (32.7) | 1 |  |
| **Respondent’s marital status** |  |  |  |
| Separated/single/widowed | 91 (11.2) | 1.50 (1.26-1.80) | 1.49 (1.15-1.94) |
| Co-habiting | 343 (41.9) | 1.34 (1.05-1.71) | 1.36 (1.02-1.80) |
| Married | 384 (46.9) | 1 | 1 |
| **Respondent’s religion** |  |  |  |
| Anglican | 234 (28.5) | 1.09 (0.84-1.41) |  |
| Catholic | 255 (31.1) | 0.88 (0.71-1.09) |  |
| Muslim | 170 (20.7) | 1.17 (0.90-1.51) |  |
| Other | 162 (19.7) | 1 |  |
| **Respondent’s occupation** |  |  |  |
| Home fulltime | 521 (63.6) | 1.60 (1.20-2.14) | 1.26 (0.87-1.83) |
| Trader | 191 (23.3) | 1.76 (1.23-2.51) | 1.14 (0.78-1.67) |
| Employed | 107 (13.1) | 1 | 1 |
| **Wealth index** |  |  |  |
| Top quintile, Least poor | 161 (20.1) | 1 | 1 |
| 4th quintile | 161 (20.1) | 1.29 (0.83-1.99) | 1.17 (0.79-1.75) |
| 3rd quintile | 152 (19.0) | 1.51 (0.97-2.37) | 1.16 (0.70-1.92) |
| 2nd quintile | 168 (20.8) | 1.51 (1.02-2.24) | 1.33 (0.87-2.05) |
| Bottom quintile, poorest | 160 (20.0) | 1.67 (1.22-2.29) | 1.38 (1.11-1.72) |
